# 

**Published:** 1893-03-04

**Authors:** 


PRICE TWOPENCE.

[No.] 336, Vol. XIII.—March 4th, 1893.

[Registered at the General Post Office as a Newspaper for transmission in the United Kingdom and Abroad.]

WITH EXTRA NURSING SUPPLEMENT.

Per Annum, 8s. 6d., post free.

Monthly Parts, 6d.; post free, 8d.

Ditto per Annum, 8s., post free.

